# Environmental Surveillance of Norovirus RNA in Restaurant Settings: Cleaning Materials as Primary Viral Reservoirs

**DOI:** 10.3390/ijerph23030321

**Published:** 2026-03-04

**Authors:** Einas A. Osman, Ahmed Al-Gafri, Abrar Albahri, Tasabih M. Saifeldin, Ayat Zawateieh, Safa. A. Abdelrahman, Emad I. Hussein

**Affiliations:** 1 College of Applied and Health Sciences, A’Sharqiyah University, Ibra 400, Oman; einas.osman@asu.edu.om (E.A.O.); azawateieh@zu.edu.jo (A.Z.); shussein5@yu.edu.jo (E.I.H.); 2 College of Human Nutrition and Dietetics, Zarqa University, Zarqa 13110, Jordan; 3 Health Information Technology Department-Medical Sterilization, King Abdulaziz University, Jeddah 21589, Saudi Arabia; sabdulalrahman@kau.edu.sa; 4 Biological Sciences Department, College of Sciences, Yarmouk University, Irbid 21163, Jordan

**Keywords:** norovirus, environmental surveillance, restaurant hygiene, viral persistence, food safety, genogroup II

## Abstract

**Highlights:**

**Public health relevance—How does this work relate to a public health issue?**

- This study addresses the prevalence of Norovirus, a leading cause of viral gastroenteritis, within the food service environment. An environmental surveillance data for Norovirus contamination in restaurants within the Gulf region.

**Public health significance—Why is this work of significance to public health?**

- Revealed a 10% contamination rate in restaurants, with all positive samples identified as the highly transmissible Genogroup II using ISO 15216-1 protocols.
- Identified cleaning materials as primary viral reservoirs, with dishcloths showing a 3-fold higher contamination rate (15%) compared to tabletops (5%).

**Public health implications—What are the key implications or messages for practitioners, policy makers and/or researchers in public health?**

- Highlights a critical need for policymakers to mandate stricter sanitation protocols, as poor cleaning practices directly correlate with higher viral detection.
- Practitioners should prioritize the management of “secondary transfer” risks, specifically moving from reusable cloths to single-use or high-disinfection cleaning methods to eliminate reservoirs.

**Abstract:**

Background: Norovirus is the leading cause of viral gastroenteritis globally, with environmental persistence contributing significantly to transmission dynamics. Despite the recognized burden in the Middle East, systematic environmental surveillance data from restaurant settings remain critically limited, particularly regarding the role of cleaning materials as reservoirs for viruses. Middle East region. Methods: A cross-sectional environmental surveillance study was conducted across 20 restaurants in Muscat and A’Sharqiyah regions, Oman (September 2020–August 2021). Forty environmental samples comprising 20 dishcloths and 20 tabletop swabs were collected from diverse restaurant types. Viral RNA was extracted using QIA amp Viral RNA MiniKit and analyzed using real-time RT-PCR following ISO/TS 15216-1:2017 protocols with genogroup-specific primers. Results: Norovirus RNA was detected in 4 of 40 samples (10%, 95% CI: 2.8–23.7%) with higher prevalence on dishcloths (3/20, 15%, 95% CI: 3.2–37.9%) versus tabletops (1/20, 5%, 95% CI: 0.1–24.9%). All positive samples were genogrouped II with cycle threshold values of 31.8–36.2. Positive samples originated from three restaurants in high-traffic urban areas, with fast-food establishments showing the highest contamination. Field observations revealed substandard 41 sanitation practices, including frequent dishcloth reuse without disinfection. Conclusions: This paper fills a gap in the current body of knowledge by offering initial systematized evidence on norovirus contamination of the environment in restaurants within the Gulf region. The results indicate that cleaning things, especially the dishwasher cloths, are the main viral reservoirs, and the contamination rates are three times higher than food-containing surfaces. These results underscore the urgent need for enhanced sanitation protocols that specifically target cleaning implements rather than surfaces alone and emphasize the importance of routine environmental surveillance in understanding and interrupting norovirus transmission dynamics in food service settings.

## 1. Introduction

Human norovirus, a member of the Caliciviridae family, represents one of the most significant foodborne pathogens globally, causing approximately 685 million cases of acute gastroenteritis annually worldwide [1]. Norovirus is the leading cause of outbreaks from contaminated food in the United States, accounting for approximately 50% of all outbreaks of food-related illness and an estimated 19–21 million illnesses annually [2]. According to the World Health Organization, the Middle East and North Africa region reported the third-highest estimated burden of foodborne diseases per population, with an estimated 70% of the region’s foodborne disease burden attributed to *E. coli*, non-typhoidal *Salmonella*, *Campylobacter*, and Norovirus [3].

The virus exhibits remarkable environmental persistence and resistance characteristics that contribute to its global prevalence. Norovirus can persist on surfaces for days or weeks and is resistant to many common disinfectants, with studies demonstrating viral survival on food-contact surfaces for up to 56 days under specific environmental conditions [4]. The extremely low infectious dose, requiring as few as 10–100 viral particles to initiate infection, combined with the ability of infected individuals to shed billions of viral particles per gram of stool or vomit, creates a highly efficient transmission pathway that is particularly challenging to control in food service environments [5,6]. Restaurants are the most common setting of foodborne norovirus outbreaks, with food workers playing a critical role in transmission dynamics. The most frequent contributing factor in restaurant outbreaks was barehand contact by a food worker suspected to be infectious (44.6%), followed by contamination by a food worker suspected to be infectious (32.6%) and gloved-hand contact by a food worker suspected to be infectious (16.3%) [7]. Environmental contamination in food service establishments poses a significant public health risk, as norovirus can retain its infectivity for extended periods on food-contact surfaces such as stainless steel and polyvinyl chloride (PVC), with persistence ranging from 7 to 56 days depending on temperature and humidity conditions [8].

Effective surveillance and detection methods are essential for understanding the extent of environmental contamination and implementing appropriate control measures. Real-time reverse transcription polymerase chain reaction (RT-PCR) represents the gold standard for norovirus detection due to the absence of reliable cell culture systems for human norovirus propagation [9,10]. The International Organization for Standardization (ISO) 15216 standard [11] provides validated protocols for quantification and detection of hepatitis A virus and norovirus in food matrices and environmental surfaces using real-time RT-PCR, ensuring standardized and reliable detection across laboratories worldwide (International Organization for Standardization) (Microbiology of the Food Chain-Horizontal Method for Determination of Hepatitis A Virus and Norovirus Using Real-Time RT-PCR-Part 1: Method for Quantification [11]. The molecular epidemiology of norovirus is characterized by genetic diversity, with genogroups I (GI) and II (GII) being the primary causes of human gastroenteritis. GII strains, particularly GII.4 variants, are responsible for the majority of global outbreaks and sporadic cases [12,13]. Recent surveillance data indicate the emergence of new variants, including GII.17, which has contributed to increased norovirus activity in recent years [14]. Understanding the prevalence and distribution of different genogroups in environmental samples provides valuable insights into transmission patterns and outbreak dynamics. Although the world has been experiencing a huge burden of the norovirus related illness, there is a great gap in the knowledge about the environmental contamination in food service outlets, especially in the Middle East region. Although foodborne infections are identified as a predominant cause of human disease in the Middle East with tough economic and population health impacts, there are few systematic surveillance studies to identify sporadic cases and outbreaks [15]. This incommensurateness of data is a hindrance to the formulation of evidence-based prevention and control strategies that are specific to regional food safety practices and environmental conditions.

There are several important roles played by the environmental monitoring of norovirus in restaurants: (i) to determine the source and mode of contamination, (ii) to assess the viability of the cleaning and disinfection measures, (iii) to shape the risk assessment and management interventions, and (iv) to offer an early warning system to any possible contamination.

The level and duration of environmental contamination have been monitored with surface sampling techniques, which have been proven effective with research indicating that norovirus is capable of surviving on a range of surfaces, including elevator buttons, toilet handles, and handrail bars [16,17].

The current research paper fills this knowledge gap by examining the level of norovirus RNA pollution on surface environments in restaurants in Oman, through the use of the standardized ISO/TS 15216-1:2017 protocol to detect and identify contamination. This study was aimed at: (1) establishing how often norovirus RNA appeared on dishcloths and tabletop surfaces in restaurants, (2) identifying the genogroups of positive samples, and (3) evaluating the connection between contamination patterns and restaurant cleaning procedures. The study can help comprehend the environmental persistence of norovirus in food service establishments and provide baseline information to conduct food safety interventions in the Gulf region.

## 2. Materials and Methods

### 2.1. Study Design and Sample Collection

**Figure 1:**
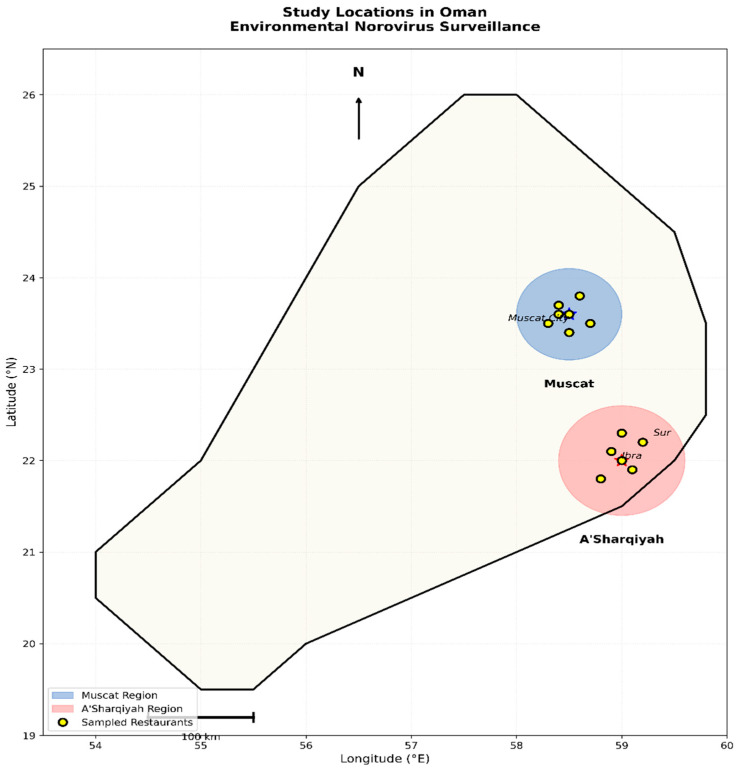
Geographic distribution of study locations. Map showing Muscat and A’Sharqiyah regions.

This cross-sectional environmental surveillance study was conducted in 20 restaurants across Muscat and A’Sharqiyah regions, Oman, from September 2020 to August 2021 (Figure 1). Restaurants were randomly selected to represent diverse food service environments: local Omani restaurants (*n* = 6), fast-food outlets (*n* = 5), Indian restaurants (*n* = 5), and Mediterranean establishments (*n* = 4). Two types of environmental samples were collected from each restaurant: dishcloths actively used by staff to wipe tables and tabletop swabs from dining areas. Dishcloths were placed directly into sterile bags, while tabletop samples were collected using sterile cotton swabs pre-moistened with sterile saline over a 100 cm^2^ surface area. All samples were transported on ice to the laboratory within 2 h.

#### 2.1.1. Restaurant Selection Criteria

Restaurants were purposively selected using the following criteria: (1) operational for minimum 6 months to ensure established cleaning practices, (2) serving minimum 50 customers daily to represent significant public health impact, (3) willingness to participate and provide verbal consent, (4) diverse geographical distribution across urban and suburban areas within study regions, (5) variety of service styles (sit-down, fast food, buffet) to capture different operational models, and (6) mix of independent and chain establishments. Restaurants were excluded if they were undergoing renovation, had reported foodborne illness outbreaks in the previous 3 months, or were temporary/seasonal establishments.

The distribution (local Omani restaurants *n* = 6, fast-food outlets *n* = 5, Indian restaurants *n* = 5, Mediterranean establishments *n* = 4) was designed to reflect the relative market prevalence of these restaurant types in the study regions. This stratified selection approach ensures representation of different food preparation practices, customer volumes, and sanitation protocols.

#### 2.1.2. Sampling Timing and Conditions

All samples were taken in the course of active operations (between 12:00–16:00 lunch service and 18:00–21:00 dinner service) to observe the contamination trends under the real food service conditions. Sampling was done at the end of shift cleaning on dishcloths in active use (1–2 h of use). The tabletops were sampled on newly wiped tables (within 515 min of wiping) without the presence of customers.

#### 2.1.3. Sample Size Justification

The study is an exploratory surveillance research that was undertaken to determine baseline prevalence estimates. The sample size (40 samples in 20 restaurants) was calculated because of logistical considerations and financial constraints rather than due to formal power considerations, since there was no available data on prevalence in the region before. This is sufficient to identify the presence of contamination rates of 15 percent and above with a power of 80 percent and alpha = 0.05, but it lacks power to identify smaller differences between surface types.

### 2.2. Sample Processing and RNA Extraction

#### 2.2.1. Detailed Sampling Protocol

Trained personnel collected all the samples in a sterile manner. In the case of tablet swabs, a 10 cm by 10 cm space was defined with the help of a sterile template. A systematic pattern was applied to sterile cotton swabs, which were pre-moistened with sterile saline (horizontal, vertical, and diagonal strokes, 10 passes each direction). In the case of dish cloths, the whole cloth was moved into sterile stomacher bags using sterile forceps.

Cross-contamination prevention measures:

1. New sterile gloves for each sample.
2. Individual packaging of all sampling materials.
3. Sterile field technique maintained throughout.
4. Samples immediately sealed and labeled.
5. Transportation in coolers with ice packs (2–8 °C).
6. Maximum 2 h transport time.
7. Laboratory processing within 4 h.
8. Field blanks included as negative controls.

#### 2.2.2. Concurrent Bacterial Enumeration

As part of the broader hygiene assessment, bacterial loads were enumerated from the same dishcloth samples. Briefly, 1 mL of the dishcloth eluate (from the stomaching process) was serially diluted in sterile PBS, and 100 μL aliquots were spread-plated onto Plate Count Agar (PCA). Plates were incubated in 35 °C in 48 h, and the total aerobic bacterial counts were determined and reported as colony-forming units per milliliter (CFU/mL). The overall indication of the microbial load and the sanitation quality was given through this enumeration, as it poses a background to the patterns of viral detection. Specific bacterial pathogen identification was not performed.

Dishcloths were processed by stomaching at 230 rpm for 2 min in 100 mL of phosphate-buffered saline (PBS, pH 7.4) [18]. Tabletop swabs were vortexed for 1 min in 10 mL sterile PBS. The resulting eluates were centrifuged at 3000× *g* for 15 min at 4 °C, and supernatants were filtered through 0.45 μm membranes to concentrate virus particles. Viral RNA was extracted from 140 μL of filtered supernatant using the QIAamp Viral RNA Mini Kit (Qiagen GmbH, Hilden, Germany) according to the manufacturer’s instructions [19]. RNA was eluted in 60 μL of RNase-free water and stored at −80 °C until analysis.

### 2.3. Real-Time RT-PCR Analysis

Viral RNA extraction and real-time RT-PCR were performed following ISO 15216-1:2017 protocols (ISO, 2017) for norovirus detection in food and environmental samples, using the (QuantiTect Probe RT-PCR Kit (QIAGEN, Hilden, Germany) [20]. Reactions were performed in 25 μL volumes containing 5 μL extracted RNA template, 12.5 μL 2× QuantiTect RT-PCR Master Mix, 0.5 μL QuantiTect RT Mix, forward and reverse primers (0.8 μM each), TaqMan probe (0.2 μM), and nuclease-free water. Genogroup-specific primers targeting the ORF1-ORF2 junction were used for norovirus genogroups I (GI) and II (GII) as specified in the ISO standard. Amplification was conducted on a StepOne Plus™ Real-Time PCR System (Applied Biosystems, Foster City, CA, USA) with the following cycling conditions: reverse transcription at 50 °C for 30 min, initial denaturation at 95 °C for 15 min, followed by 45 cycles of denaturation at 95 °C for 15 s and annealing/extension at 60 °C for 60 s.

### 2.4. Quality Control and Data Interpretation

#### 2.4.1. Comprehensive Quality Control Measures

Extraction Controls: Process controls: Mouse norovirus RNA spiked into negative samples to monitor extraction efficiency (recovery rate: 85–95%).

Negative extraction controls: Sterile PBS processed alongside samples to detect reagent contamination.

Positive extraction controls: Known norovirus-positive samples to verify protocol efficacy.

#### 2.4.2. PCR Controls

Positive amplification controls: Synthetic norovirus GI and GII RNA (Eurofins Genomics, Ebersberg, Germany) run in duplicate (expected Ct: 25–30).

Negative amplification controls: RNase-free water in place of template.

Internal amplification control: GAPDH gene to verify absence of PCR inhibitors.

#### 2.4.3. Cross-Contamination Prevention

- Separate designated areas for sample processing, RNA extraction, and PCR setup.
- Unidirectional workflow to prevent amplicon contamination.
- UV irradiation of work surfaces between samples.
- Aerosol-resistant pipette tips throughout.
- Regular decontamination with RNase Away.
- Field blanks to monitor sampling contamination.

#### 2.4.4. Positivity Criteria and Threshold Justification

Samples were considered positive with Ct values ≤ 40, following ISO/TS 15216-1:2017 recommendations. This threshold is supported by: (1) environmental samples contain lower viral concentrations than clinical specimens, necessitating higher sensitivity, (2) validation studies show Ct values up to 40 correlates with culturable virus in surrogate systems, (3) norovirus’s extremely low infectious dose means even high Ct values may represent public health significance, and (4) this threshold maintains consistency with international surveillance programs.

### 2.5. Statistical Analysis

Descriptive statistics were calculated for all variables. Detection rates were expressed as proportions with [95% confidence intervals calculated using the Wilson score method (appropriate for small sample sizes and proportions near boundaries)] ← RED. Differences in contamination prevalence between sample types (dishcloths vs. tabletops) and restaurant categories were assessed using Fisher’s exact test (two-tailed), [selected due to small expected cell counts (<5) in contingency tables that violate chi-square assumptions] ← RED. [Cycle threshold values among positive samples were summarized using median and range, given the small number of positive samples (*n* = 4)] ← RED. Statistical significance was set at α = 0.05. All analyses were performed using IBM SPSS Statistics Version 16.0 (IBM Corp., Armonk, NY, USA). [For bacterial count comparison, log-transformed CFU/mL values were compared using the Mann–Whitney U test, given non-normal distribution].

### 2.6. Genogroup Identification

Positive samples were characterized by genogroup (GI or GII) based on the specific primer sets that yielded positive amplification results.

### 2.7. Ethical Considerations

Even though human subjects were not used in this research, ethical considerations were taken into account during the research process. Before the collection of samples, restaurant managers were given verbal consent, which was transparent and voluntary. All data analysis and reporting were performed anonymously by avoiding the identities of the respective restaurants, and there was no record or disclosure of any identifying information.

## 3. Results

### 3.1. Norovirus RNA Detection and Genogroup Analysis

**Figure 2:**
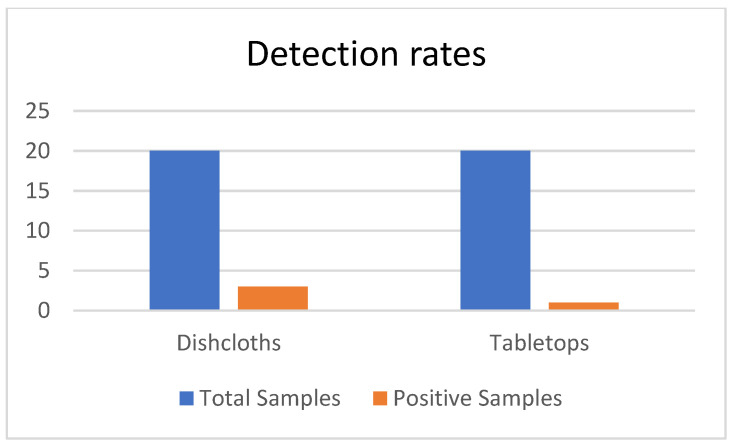
Norovirus RNA detection rates.

Norovirus RNA was detected by real-time RT-PCR in 4/40 samples (10 percent, 95 percent interval: 2.8–23.7 percent), with significant differences among different types of tablets: numerically higher in dishcloths (3/20, 15 percent, 95 percent interval: 3.2–37.9 percent) than in tabletops (1/20, 5 percent, 95 percent interval: 0.1–24.9 percent). Nevertheless, the difference was not statistically significant according to the Fisher exact test (p = 0.342), as this is exploratory research and the statistics is limited because of the limited sample size. The wide confidence intervals underscore the need for larger-scale surveillance to establish precise prevalence estimates (Figure 2). All positive samples were identified as genogroup II (GII), consistent with global epidemiological patterns. Cycle threshold values ranged from 31.8 to 36.2, indicating low-to-moderate viral RNA loads. Important limitation: RT-PCR detects viral RNA and cannot distinguish between infectious and non-infectious viral particles. The detected RNA may represent intact infectious virions, degraded non-infectious virus, or a mixture of both. However, the Ct values observed suggest potential public health relevance when considered alongside norovirus’s low infectious dose and environmental stability characteristics.

Norovirus RNA detection rates by surface type are demonstrated in Table 1 resulting in 3 samples in dishcloths compared to 1 sample in tabletops suggesting that dishcloths may represent a higher-risk surface for viral persistence and potential cross-contamination. Grouped bar chart comparing detection rates between dishcloth samples (*n* = 20) and tabletop swab samples (*n* = 20) collected from 20 restaurants in Muscat and A’Sharqiyah regions, Oman (September 2020–August 2021). For each surface type, blue bars represent total samples analyzed, while red bars represent samples testing positive for norovirus RNA by RT-PCR. Detection percentages are labeled above bars: 15% (3/20 samples) for dishcloths and 5% (1/20 samples) for tabletops. Error bars represent 95% confidence intervals calculated using the Wilson score method. All positive samples were identified as genogroup II (GII). While dishcloths demonstrated three-fold higher numerical detection rate, this difference was not statistically significant (*p* = 0.342, Fisher’s exact test), reflecting the exploratory nature of this pilot study.

As shown in Table 2, fast-food establishments demonstrated the highest restaurant-level contamination rate (40%), followed by Omani restaurants (16.7%), while no contamination was detected in Indian or Mediterranean establishments. When analyzed by sample type within positive restaurants, dishcloths consistently showed higher contamination than tabletops across all positive establishments.

Table 3 shows that restaurants reporting positive results in Norovirus, demonstrated consistently higher bacterial loads (>10^5^ CFU/mL), whereas the others revealed lower bacterial levels. This observational correlation suggests that poor general hygiene practices may predispose establishments to multiple microbial contamination types. However, causative relationships cannot be established from this cross-sectional statistically significant data (*p* = 0.342) due to the limited sample size.

Distribution of norovirus contamination by restaurant type. Bar chart showing norovirus detection rates across four restaurant categories: local Omani restaurants (*n* = 6), fast-food outlets (*n* = 5), Indian restaurants (*n* = 5), and Mediterranean establishments (*n* = 4) sampled in Muscat and A’Sharqiyah regions, Oman. For each category, gray bars display the total number of restaurants sampled, while red bars show the number of restaurants with at least one positive sample. Restaurant-level detection percentages are labeled above each group. Fast-food establishments showed the highest contamination, with 2 of 5 restaurants (40%) yielding positive samples, followed by Omani restaurants with 1 of 6 (16.7%). No contamination was detected in Indian or Mediterranean establishments. Within positive restaurants, dishcloths consistently showed higher contamination than tabletops (data shown in Figure 2), indicating cleaning materials as primary viral reservoirs across restaurant types.

### 3.2. Contamination Distribution and Quality Control

**Figure 3:**
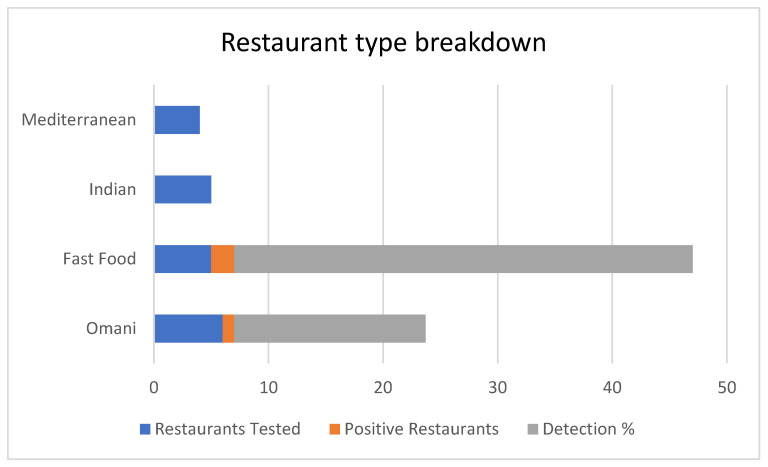
Restaurant type breakdown.

Positive samples originated from three different restaurants in high-traffic urban areas with observed substandard sanitation practices, including infrequent dishcloth replacement and inadequate disinfection. Fast-food establishments yielded the highest contamination rates, while no positive samples were detected in Mediterranean restaurants (Figure 3). All quality control measures performed satisfactorily: positive controls showed consistent amplification (Ct 25–30), negative controls remained undetected, and process controls demonstrated successful RNA extraction without significant inhibition. Statistical analysis using Fisher’s exact test showed numerically higher detection in dishcloths versus tabletops, though not.

### 3.3. Cross-Correlation with Contamination Patterns

Comparison to concurrent data on bacterial contamination indicated that significant correlations were observed: restaurants that were found to have norovirus positive samples also had high bacterial loads (>10^5^ CFU/mL on dishcloths) and Mediterranean restaurants with lowest bacteria contamination also had no norovirus detections. This relationship indicates that inadequate general hygiene habits precondition the establishments with various forms of microbial contamination. The moderately high Ct values (31.8–36.2), represent viral loads that have the potential to represent infectious particles, with norovirus being environmentally stable and having low infectious doses of the virus to produce infection.

## 4. Discussion

The current research presents the initial systematic evaluation of the norovirus environmental contamination in Oman-based restaurant establishments, which offers valuable information on the nature of viral persistence and transmission routes. The fact that norovirus RNA is detected in 10% of environmental samples, although this prevalence is significantly higher in dishcloths (15%) than in tabletops (5%), implies the importance of cleaning materials in the spread of viruses. The prevalence of the GII genogroup II (GII) strains is also in line with the global epidemiological trends, with GII.4 variants being the cause of most norovirus outbreaks in the world [13,14,21,22].

The cycle threshold values (31.8–36.2) are low to moderate viral loads, which represent contamination of surfaces of the environment as opposed to direct fecal deposition. Although these would be considered relatively low viral concentrations, past research shows that norovirus can still be infectious at very low viral concentrations compared to other enteric viruses, and as few as 10,100 viral particles can be infectious [5,6]. This very low infectious dose implies that even moderate levels of viruses’ present may pose a serious threat to the overall health of a population in case it is transmitted to food or ingested via hand-to-mouth contact [23].

One of the inherent shortcomings of RT-PCR-based environmental surveillance is the fact that it is impossible to conclusively determine the existence of an infectious virus. Our molecular approach detects viral genetic material but cannot determine viral viability. The detected RNA may represent: (1) intact infectious virions capable of initiating infection, (2) damaged virions with intact RNA but compromised infectivity, (3) free RNA released from degraded viral particles, or (4) a combination of these scenarios.

However, several contextual factors suggest potential transmission risk from our positive samples. Environmental samples from actively used cleaning materials likely contain recently deposited virus rather than degraded residual RNA. Norovirus demonstrates exceptional environmental stability, maintaining infectivity on surfaces for extended periods (up to 28 days on stainless steel under optimal conditions). Correlation studies with cultivable surrogate viruses suggest that environmental samples with Ct < 38 frequently contain viable virus. Most importantly, the extremely low infectious dose (10–100 particles) means even partially viable populations pose transmission risk.

Future methodological improvements should address the infectivity question. Viability RT-qPCR using propidium monoazide (PMA) or ethidium monoazide (EMA) pretreatment selectively detects intact capsids [24]. The actual norovirus cultivation is now possible in human intestinal enteroid (HIE) culture systems, although these are technically difficult [25]. The integrity of the capsid can be used to distinguish between intact and damaged virions [23]. Next-generation surveillance studies should focus on these sophisticated methodologies to better determine social health risks due to environmental pollution [24,25].

The tripled rate of detection in dishcloths is one of the strongest findings, and it presents quantitative data on the significance of cleaning materials as the main source of viruses. This is in agreement with previous studies, which indicate that dishcloths always have higher loads of microbes than their clean surfaces [16,26]. High-quality viral recovery of dishcloths is likely to represent the best environments to retain viruses: dampness, presence of organic material, and anti-desiccation. The dishcloths that contain viable virus particles act as an efficient transmission vehicle, causing contamination spread across various surfaces during the period of use. Observations in the field have shown that dishcloths were often used on various surfaces without being washed in-between and this provided direct routes of cross-contamination in the dining and food preparation zones.

Our results indicate that various causes of the high levels of dishcloth contamination were found. The main sources of contamination can be identified as: (1) Hand contamination- food handlers who are infected with no symptoms or in pre-symptomatic stages might be direct carriers of the virus to cloths during hand-handling. Research has shown that 2030% of food handlers can be asymptomatic shedders [1,5]. (2) Environmental transfer-residential clothes worn to cleanse contaminated surfaces obtain and accumulate viral particles of various sources during the service episode. (3) Contaminated rinse water- reuse of rinse water or lack of enough water exchange will permit the concentration of viruses in the aqueous phase, and consequent transfer to cloths.

Contamination is further aggravated by amplification and persistence factors. Poor disinfection was also observed; with the naked eye, it was seen that the majority of the establishments were only rinsed using water, or general-purpose detergent without virucidal disinfectants (chlorine-based or quaternary ammonium at the right concentration) to clean the cloths [27,28]. Moisture retention—the consistently moist environment of dishcloths prevents desiccation-mediated viral inactivation, thus staying infectious over long intervals [29,30]. Prolonged use—single cloth usage of 2–4 h without replacement makes viral accumulations and concentration possible. Poor separation- a single cloth is used in various tasks (cleaning tables, wiping counters, wiping equipment), which promotes cross-contamination.

These results imply that successful intervention should be a multi-faceted process that addresses the causes of contamination, disinfection procedures, and practices as opposed to a single control measure.

Positive samples were collected in restaurants of high-traffic urban centers in which poor sanitation habits were observed. The high contamination of fast-food restaurants was also accompanied by high levels of bacterial loads in parallel tests, indicating that inadequate general hygiene practices are the cause of predisposition to various types of microbial contamination in establishments. On the other hand, low contamination of bacteria among Mediterranean restaurants also did not reveal norovirus, which supports the correlation between the overall sanitation levels and pathogen control. This shows that global hygiene interventions can be more constructive than pathogen-specific ones.

The Oman food safety regulatory system, which is controlled by the Ministry of Agriculture, Fisheries and Water Resources (MAFWR) and the Public Authority of Consumer Protection (PACP), stipulates compulsory food service establishments standards depending on the Gulf Cooperation Council (GCC) standardization principles. Regulations are as follows: (1) clean and sanitized cloths or single-use surfaces are to be used to clean surfaces; (2) cloths used to clean surfaces during service should be separated; (3) single-use clean and sanitized cloths or single-use surfaces; (4) replacement of certain cloths during service at least after 2 h every time.

The behavior in restaurants that have tested positive in norovirus such as the longer use of cloths (>3 h), lack of sanitizer buckets, and wearing the same cloth on different surfaces are the clearer breaks of these established standards. This evidence indicates that regulatory frameworks are in place, but inspection and compliance are inadequate, which is why inspection measures must be improved and food handler training programs that will address the issue of viral pathogen control measures in particular [31].

The noted association between increased bacterial carriage and norovirus identification, albeit not being causal, indicates that a systemic hygiene intervention in terms of general hygiene conditions could prove to be more efficient than pathogen-specific interventions. The bacterial indicators (total aerobic counts) may be used as a possible screening tool to identify the high-risk establishments that should be provided with specific viral surveillance but it would not be specific due to the strong differences in the sources of bacterial and viral contamination as well as their persistence properties.

Environmental pollution is a long-term source of transmission beyond acute disease in food handlers. The long-lasting staying time (days–weeks) of norovirus in normal restaurant environments presents a delayed source of transmission in which the contamination of an infected person leads to other people becoming sick even after the event has occurred. The overwhelming percentage of dishcloth contamination demonstrates the shortcomings of the current hand hygiene measures, which focus on the cleanliness of the surface and reduction of bacteria but fail to consider the necessity of eliminating the virus.

The 10% rate of environmental detection is within the range reported on the same studies in other areas, but again, cross-comparisons should be made with caution to methods. Studies from East Asia report environmental norovirus prevalence of 5–18% in food service settings during non-outbreak periods, with Japan reporting 12% detection in restaurant kitchen environments and South Korea detecting norovirus in 8% of food-contact surfaces [32]. European surveillance data indicate 7–15% detection rates in routine restaurant monitoring, with higher rates (20–35%) during winter peak seasons. Studies conducted in the United States have reported environmental norovirus detection rates ranging from 5 to 25% in food service settings, with higher rates typically observed during outbreak periods or in establishments with recent illness reports.

Limited data from the Middle East region make our study particularly valuable; a single study from Turkey reported 6% detection [33,34], while no published data exist from the Gulf Cooperation Council (GCC) countries before this investigation. Our finding that dishcloths harbor higher contamination than surfaces align with international observations from the United Kingdom and the United States, where cleaning materials consistently show 2–4-fold higher detection rates compared to food-contact surfaces. GII.4 variants represent 60–80 per cent of norovirus identifications in the world [32,35], which explains the over-representation of GII strains in our samples. These consistencies indicate that the dynamics of contamination that we have identified in Omani restaurants are reflective of broader trends in norovirus environmental persistence that do support the generalizability of our results and indicate that GI strains may have distinct environmental persistence mechanisms or were simply below the detection limit of the assay.

In this study, the standardized ISO protocols of norovirus detection were used that guaranteed the rigor of methods and global comparability of findings. The real-time RT-PCR of the ORF1-ORF2 junction is sensitive and specific to the detection of noroviruses, whereas the characterization of the epidemiologically relevant strains is possible with the help of genogroup-specific primers. The addition of suitable positive and negative controls as well as process controls to determine possible PCR inhibition enhances the trust in the analysis outcome. A number of significant limitations will have to be admitted, which influence the interpretation and generalization of our findings.

Sample Size and Statistical Power: The small sample size (40 samples of 20 restaurants) restricts the extrapolation of our 10 percent prevalence estimate to the rest of the Gulf or other types of restaurants that were not in our sample. This research ought to be viewed as an exploratory study in nature, where it will be able to give baseline surveillance data, but not an actual prevalence estimate. The low level of statistical power is reflected when the difference between the use of dishcloths and tabletops is not significant (p = 0.342), though the numerical difference is three times. The large confidence intervals of our prevalence estimates (95% CI: 2.8–23.7% overall detection) do not allow accurate regional extrapolation. In future research, the larger sample sizes should be used (stratified random samples within larger geographic areas and other restaurant catteries) to come up with stronger prevalence estimates with smaller confidence intervals.

Surface Type Limitations: Our sampling approach did not target a full range of environmental contamination by dishcloths and tabletops. Other high-touch surfaces, such as door handles, menu cards, payment terminals, restroom fixtures, and handrails, might provide other sources of the virus and be the focus of war-related research in future extensive surveillance efforts.

Temporal Snapshot: The single-time-point sampling design measures prevalence at the time of collection but does not measure temporal dynamics, seasonal patterns, or the persistence of contamination across time. A longitudinal sampling across the seasons and across the working periods would yield a more detailed insight into the contamination trends.

RNA vs. Infectious Virus: RT-PCR will identify viral nucleic acid and will not definitely identify the presence of infectious viral particles, as explained above. Although according to our Ct values, we have possibilities of indicating potential infectivity based on a correlation study, we have to determine the infectivity with complementary means using cell culture or a molecular viability test.

Qualitative Observations: The observations of the sanitation practices were qualitative field notes and not quantitative measurements with validated instruments. In future studies, the validated sanitation assessment instruments should be included, including the WHO Environmental Health Assessment Tool or FDA Food Code compliance checklists [36], so that the quantitative correlation analysis between individual hygienic parameters and the level of viral contamination will be possible.

Depending on our cost of study, RT-PCR-based surveillance such as sample collection, RNA extraction and real-time PCR analysis costs about 25–30 USD per sample at large-scale. Quarterly sampling of 2–3 surfaces per restaurant of a risk-based surveillance program focuses on high-risk establishments (large number of customers served, history of past violations, complicated menu), using the selected establishments as the target population, would cost 200–360 USD annually per establishment. This is 0.1 percent of the operating expenses in medium to large restaurants.

There is an indication of good economics in cost-benefit analysis. The cost of average norovirus outbreaks in food service facilities is 5000–50,000 USD (lost revenue, cleaning, damaged reputation, possible legal expenses) [1,32]. One prevented outbreak equals 14–500 annual surveillance costs of establishments. Others are improved food safety culture, lower liability and possible marketing advantage.

Feasibility considerations include: (1) Laboratory capacity: The local laboratory should have reference laboratories with RT-PCR and trained staff. (2) Sample logisticians require cold chain and fast delivery systems. (3) Turnaround time- results that must be obtained within 48–72 h to take action. (4) Integration of regulatory structures with the currently established inspection systems.

Implementation advice: The most viable approach seems to be a tiered approach [31,36]. Tier 1 (High-risk): Surveillance (quarterly) of high-volume restaurants, catering, and institutional foodservice. Tier 2 (Medium-risk): Medium-volume establishments surveyed every 2 years. Tier 3 (Low-risk): Surveillance per annum or triggered by complaint/outbreak. Efficient use of surveillance to maximize effect [32,37]. Other quicker techniques (immunochromatographic testing, portable PCR) that are being developed can further lower the cost and enhance the possibility of a wide implementation of it.

### Practical Recommendations for Norovirus Outbreak Surveillance

Using our results, we suggest the following feasible outline of emergency norovirus surveillance in the restaurant environment [31,36]:

Immediate Response (0–24 h): Take standardized dish cloths and high-touch surface swabs in the implicated set-ups. Introduce safer sanitization using effective virucidal agents (sodium hypochlorite at a concentration of 1000–5000 ppm or more). Put a temporary ban on the use of reusable cloth until the assessment of contamination is done. Isolate symptomatic food handlers and use extreme hand hygiene precautions.

Short-term Actions (1–7 days): RT-PCR analysis is performed based on the ISO 15216-1 protocols [11] with 24 to 48 h turnaround results. Increase the number of samples with food handler hand swabs and food-contact surfaces. Begin epidemiological research connecting environmental findings with the case data. Identify single-use cleaning materials or increased disinfection measures as a mandatory measure. Carry out food handler training on transmission and prevention of norovirus. Long-term Surveillance (continuous): Have regular environmental surveillance programs in the high-risk places of work [1,38]. Introduce quarterly sampling of cleaning products at food service agencies. Establish regional laboratory capacity on rapid detection of norovirus [25,39]. Combine data on environmental monitoring and clinical reporting of cases. Set threshold triggers on improved surveillance or temporary shutdown. Develop feedback systems to update food safety policy and regulation.

## 5. Conclusions

This study is the first to provide systematic evidence of norovirus environmental contamination within restaurants in this Gulf region, with a baseline detection rate of 10 percent and identification of cleaning materials as the most important viral reservoirs, with three times more contamination on food-contact surfaces. The only genogroup II helped to prove that widely spread strains can still be found in a local food service environment, and presenting indicate that dishcloths can be a more dangerous surface for viral persistence and potential cross-contamination.

The implications in practice are considerable: the existing sanitation guidelines, which emphasize cleaning surfaces and neglect cleaning tools, can be used unknowingly to contribute to the spread of the virus. The findings can be used by food safety regulators in Oman and the rest of the Gulf region to justify several regulatory changes: mandatory switching to single-use cleaning materials or reusable item virucidal disinfection, introduction of regular environmental testing programs in high-risk businesses, increased focus on food handler training (particularly addressing the persistence and transmissibility of norovirus), and establishment of regional laboratory networks to support rapid environmental surveillance to prevent and respond to outbreaks.

In addition to the regional setting, this study will add methodological precedent and baseline figures that allow cross-regional comparisons, as well as evidence-based food safety policies that are on a global level. Above all, the results show that environmental surveillance could define patterns of contamination and risks of transmission before the start of the outbreak, which is a vital contribution to preventing the ensuing outbreak through proactive prevention measures, and not the reactive response to outbreaks, which is the enhancement of the food safety conditions in restaurants.

## Figures and Tables

**Table 1:** Summary of norovirus detection results.

Sample Type	Total Samples	Positive Samples	Detection Rate (%)	Ct Value Range
Dishcloths	20	3	15.0	31.8–35.7
Tabletops	20	1	5.0	36.2
Total	40	4	10.0	31.8–36.2

All positive samples were identified as norovirus genogroup II (GII). Ct = cycle threshold.

**Table 2:** Norovirus detection by restaurant type.

Restaurant Type	Restaurants Tested	Positive Restaurants	Detection Rate (%)
Omani	6	1	6.7
Fast Food	5	2	40.0
Indian	5	0	0.0
Mediterranean	4	0	0.0
Total	20	3	15.0

**Table 3:** Bacterial loads and norovirus detection in dishcloth samples.

Sample Category	Bacterial Count Range (CFU/mL)	Norovirus Detection
Norovirus-positive	1.2 × 10^5^–3.4 × 10	Detected (*n* = 3)
Norovirus-negative	8.2 × 10^3^–1.1 × 10^5^	Not detected (*n* = 17)

## Data Availability

The raw data supporting the conclusions of this article will be made available by the authors upon request.

## References

[1] Hall A. (2014). Projecting regional change. Science.

[2] AMA (2025). Proceedings of the 2025 Annual Meeting of the House of Delegates.

[3] WHO (2007). WHO Estimates of the Global Burden of Foodborne Diseases.

[4] Lamhoujeb S., Fliss I., Ngazoa S.E., Jean J. (2009). Molecular Study of the Persistence of Infectious Human Norovirus on Food-Contact Surfaces. Food Environ. Virol..

[5] Teunis P.F.M., Moe C.L., Liu P., EMiller S., Lindesmith L., Baric R.S., Le Pendu J., Calderon R.L. (2008). Norwalk virus: How infectious is it?. J. Med. Virol..

[6] Atmar R.L., Opekun A.R., Gilger M.A., Estes M.K., Crawford S.E., Neill F.H., Graham D.Y. (2008). Norwalk Virus Shedding after Experimental Human Infection. Emerg. Infect. Dis..

[7] Hoover E.R., Hedeen N., Freeland A., Kambhampati A., Dewey-Mattia D., Scott K.-W., Hall A., Brown L. (2020). Restaurant Policies and Practices Related to Norovirus Outbreak Size and Duration. J. Food Prot..

[8] Arthur S.E., Gibson K.E. (2016). Environmental persistence of Tulane virus—A surrogate for human norovirus. Can. J. Microbiol..

[9] Bustin S.A., Benes V., Garson J.A., Hellemans J., Huggett J., Kubista M., Mueller R., Nolan T., Pfaffl M.W., Shipley G.L. (2009). The MIQE Guidelines: Minimum Information for Publication of Quantitative Real-Time PCR Experiments. Clin. Chem..

[10] Kageyama T., Kojima S., Shinohara M., Uchida K., Fukushi S., Hoshino F.B., Takeda N., Katayama K. (2003). Broadly Reactive and Highly Sensitive Assay for Norwalk-Like Viruses Based on Real-Time Quantitative Reverse Transcription-PCR. J. Clin. Microbiol..

[11] (2017). Microbiology of the Food Chain-Horizontal Method for Determination of Hepatitis A Virus and Norovirus Using Real-Time RT-PCR. Part 1: Method for Quantification.

[12] Robilotti E., Deresinski S., Pinsky B.A. (2015). Norovirus. Clin. Microbiol. Rev..

[13] Vinjé J. (2015). Advances in Laboratory Methods for Detection and Typing of Norovirus. J. Clin. Microbiol..

[14] Cannon J.L., Barclay L., Collins N.R., Wikswo M.E., Castro C.J., Magaña L.C., Gregoricus N., Marine R.L., Chhabra P., Vinjé J. (2019). Correction for Cannon et al., “Genetic and Epidemiologic Trends of Norovirus Outbreaks in the United States from 2013 to 2016 Demonstrated Emergence of Novel GII.4 Recombinant Viruses”. J. Clin. Microbiol..

[15] Osaili T.M., Obeidat B.A., Abu Jamous D.O., Bawadi H.A. (2011). Food safety knowledge and practices among college female students in north of Jordan. Food Control.

[16] Barker J., Vipond I.B., Bloomfield S.F. (2004). Effects of cleaning and disinfection in reducing the spread of Norovirus contamination via environmental surfaces. J. Hosp. Infect..

[17] Gallimore C.I., Cheesbrough J.S., Lamden K., Bingham C., Gray J.J. (2005). Multiple norovirus genotypes characterised from an oyster-associated outbreak of gastroenteritis. Int. J. Food Microbiol..

[18] Kazama S., Miura T., Masago Y., Konta Y., Tohma K., Manaka T., Liu X., Nakayama D., Tanno T., Saito M. (2017). Environmental Surveillance of Norovirus Genogroups I and II for Sensitive Detection of Epidemic Variants. Appl. Environ. Microbiol..

[19] Qiagen (2020). QIAamp Viral RNA Mini Handbook.

[20] Qiagen (2018). QuantiTect Probe RT-PCR Handbook.

[21] Hirneisen K.A., Kniel K.E. (2013). Comparing Human Norovirus Surrogates: Murine Norovirus and Tulane Virus. J. Food Prot..

[22] Manuel C.S., Moore M.D., Jaykus L.-A. (2018). Predicting human norovirus infectivity—Recent advances and continued challenges. Food Microbiol..

[23] Randazzo W., Truchado P., Cuevas-Ferrando E., Simón P., Allende A., Sánchez G. (2020). SARS-CoV-2 RNA in wastewater anticipated COVID-19 occurrence in a low prevalence area. Water Res..

[24] Fraisse A., Niveau F., Hennechart-Collette C., Coudray-Meunier C., Martin-Latil S., Perelle S. (2018). Discrimination of infectious and heat-treated norovirus by combining platinum compounds and real-time RT-PCR. Int. J. Food Microbiol..

[25] Knight V., McKissick B.R., Saunders A. (2013). A Review of Technology-Based Interventions to Teach Academic Skills to Students with Autism Spectrum Disorder. J. Autism Dev. Disord..

[26] Scott E., Bloomfield S.F. (1990). The survival and transfer of microbial contamination via cloths, hands and utensils. J. Appl. Bacteriol..

[27] Liu E., Yan C., Mei X., He W., Bing S.H., Ding L., Liu Q., Liu S., Fan T. (2010). Long-term effect of chemical fertilizer, straw, and manure on soil chemical and biological properties in northwest China. Geoderma.

[28] Park S.-H., Kwak T.-K., Chang H.-J. (2010). Evaluation of the food safety training for food handlers in restaurant operations. Nutr. Res. Pract..

[29] Rzeżutka A., Cook N. (2004). Survival of human enteric viruses in the environment and food. FEMS Microbiol. Rev..

[30] Yeargin S.W., Dompier T.P., Casa D.J., Hirschhorn R.M., Kerr Z.Y. (2019). Epidemiology of Exertional Heat Illnesses in National Collegiate Athletic Association Athletes During the 2009–2010 Through 2014–2015 Academic Years. J. Athl. Train..

[31] Barclay S., Froggatt K., Crang C., Mathie E., Handley M., Iliffe S., Manthorpe J., Gage H., Goodman C. (2014). Living in uncertain times: Trajectories to death in residential care homes. Br. J. Gen. Pract..

[32] Verhoef P.C., Kannan P.K., Inman J.J. (2015). From Multi-Channel Retailing to Omni-Channel Retailing. J. Retail..

[33] Kamel A.H., Ali M.A., El-Nady H.G., de Rougemont A., Pothier P., Belliot G. (2009). Predominance and Circulation of Enteric Viruses in the Region of Greater Cairo, Egypt. J. Clin. Microbiol..

[34] Sdiri-Loulizi K., Gharbi-Khélifi H., de Rougemont A., Chouchane S., Sakly N., Ambert-Balay K., Hassine M., Guédiche M.N., Aouni M., Pothier P. (2008). Acute Infantile Gastroenteritis Associated with Human Enteric Viruses in Tunisia. J. Clin. Microbiol..

[35] Karst S.M., Zhu S., Goodfellow I.G. (2015). The molecular pathology of noroviruses. J. Pathol..

[36] O’Grady N.P., Alexander, Burns L.A., Dellinger P., Garland, Heard S.O., Lipsett P.A., Masur, Mermel L.A., Pearson M.L. (2011). Guidelines for the Prevention of Intravascular Catheter-Related Infections. Clin. Infect. Dis..

[37] Rockx B.H.G., Vennema H., Hoebe C.J.P.A., Duizer E., Koopmans M.P.G. (2005). Association of Histo–Blood Group Antigens and Susceptibility to Norovirus Infections. J. Infect. Dis..

[38] Mattison K., Shukla A., Cook A., Pollari F., Friendship R., Kelton D., Bidawid S., Farber J.M. (2007). Human Noroviruses in Swine and Cattle. Emerg. Infect. Dis..

[39] Baert L., Mattison K., Loisy-Hamon F., Harlow J., Martyres A., Lebeau B., Stals A., Van Coillie E., Herman L., Uyttendaele M. (2011). Review: Norovirus prevalence in Belgian, Canadian and French fresh produce: A threat to human health?. Int. J. Food Microbiol..

